# Super Resolution Microscopy of SUMO Proteins in Neurons

**DOI:** 10.3389/fncel.2019.00486

**Published:** 2019-11-01

**Authors:** Luca Colnaghi, Luca Russo, Carmina Natale, Elena Restelli, Alfredo Cagnotto, Mario Salmona, Roberto Chiesa, Luana Fioriti

**Affiliations:** ^1^Department of Neuroscience, Dulbecco Telethon Institute, Istituto di Ricerche Farmacologiche Mario Negri IRCCS, Milan, Italy; ^2^Department of Molecular Biochemistry and Pharmacology, Istituto di Ricerche Farmacologiche Mario Negri IRCCS, Milan, Italy; ^3^Department of Neuroscience, Istituto di Ricerche Farmacologiche Mario Negri IRCCS, Milan, Italy

**Keywords:** small ubiquitin-like modifier, neuron, synapse, super resolution microscopy, synaptophysin, PSD95

## Abstract

The ubiquitously expressed SUMO proteins regulate a plethora of cellular pathways and processes. While they have a predominantly nuclear localization, extranuclear roles of SUMO isoforms at the synapse have also been described, making SUMOylation one of the major post-translational regulators of nerve functions. These findings have however recently been challenged, at least for SUMO1, by the analysis of knock-in mice expressing His_6_-HA-SUMO1, where the authors failed to detect the protein at the synapse. In the ongoing dispute, the subcellular distribution in neurons of SUMO2/3 and of the E2 SUMO ligase Ubc9 has not been examined. To investigate whether SUMO proteins do or do not localize at the synapse, we studied their localization in hippocampal primary neurons by super resolution microscopy. We found that SUMO1, SUMO2/3, and Ubc9 are primarily nuclear proteins, which also colocalize partially with pre- and post-synaptic markers such as synaptophysin and PSD95.

## Introduction

Small ubiquitin-like modifier (or SUMO) proteins are similar to ubiquitin and are considered members of the ubiquitin-like protein family. Mammals express five different SUMO variants: SUMO1-5. All variants share a similar size of about 11 kDa and an almost identical three-dimensional structure. The first SUMO variant to be identified was SUMO1 in 1997 (Johnson et al., 1997), followed by the discovery of SUMO2 and 3 by homology screening. SUMO2 and 3 differ by only 3 amino acids and they are often considered identical and referred to as SUMO2/3. Finally, SUMO4 and SUMO5 have been proposed to be two additional members of the SUMO family (Liang et al., 2016; Cappadocia and Lima, 2018). Like ubiquitin, SUMO proteins can be covalently conjugated to lysine residues of target proteins by an enzymatic cascade, shared by all SUMO proteins, that closely resembles that used by the ubiquitination machinery. Differently from ubiquitin, however, where the variety of E1, E2, and E3 enzymes is great (as well as their possible combinations), mammals only encode one SUMO-E1 enzyme (the heterodimer SAE1/SAE2), one SUMO-E2 enzyme (Ubc9), and a dozen of SUMO-E3 enzymes (Gareau and Lima, 2010). SUMO itself does not have any enzymatic activity, and its conjugation to proteins may exert the following functions: (i) modulate target stability; (ii) induce conformational changes to regulate the target function; (iii) reduce or induce protein-protein interactions; or (iv) affect the cellular localization of the target (Flotho and Melchior, 2013; Cappadocia and Lima, 2018). All these effects are not mutually exclusive and can influence a multitude of cellular functions, from gene expression to DNA repair, from protein trafficking to synaptic plasticity (Droescher et al., 2013; Guo and Henley, 2014).

Evidence in support of a synaptic role comes primarily from studies on SUMO1, which has been detected in synaptic fractions, it has been shown to co-localize with synaptic markers and to be conjugated to synaptic proteins to regulate synaptic activity (Henley et al., 2018). Data suggesting the presence of SUMO2/3 and SUMOylation enzymes at the synapse is less abundant. In 2004, an unbiased proteomic analysis of rat brain postsynaptic density found SUMO2 as the only SUMO variant in the extracts (Li et al., 2004). In 2012 and 2014, the SUMOylation enzyme Ubc9 was found to be enriched in dendritic spines (Loriol et al., 2012, 2014). In 2015, we described the protein CPEB3 to be SUMOylated by SUMO2 in synaptosomal fractions (Drisaldi et al., 2015). Finally, Ghosh et al. (2016) reported that gephyrin, an essential scaffolding protein at GABAergic synapses, is modified by both SUMO1 and SUMO2. Recently however, the presence and role of SUMO at the synapse have been challenged by a new set of studies primarily using a knock-in mouse expressing a His_6_-HA-SUMO1 transgene, a panel of SUMO1-specific antibodies and SUMO1 knock-outs as control animals (Daniel et al., 2017, 2018). In these studies, the authors failed to detect SUMO1 at the synapse and SUMOylation by SUMO1 of target proteins in synaptosomal preparations. In this dispute, very little attention has been paid to SUMO2/3 and to SUMOylation enzymes such as Ubc9 (Wilkinson et al., 2017; Daniel et al., 2018). In order to clarify whether SUMO and SUMO-related proteins are present at the synapse, we analyzed endogenous SUMO2/3, Ubc9 and SUMO1 in hippocampal neuronal cultures by immunofluorescence super resolution microscopy, using at least two anti-SUMO1, SUMO2/3, and Ubc9 antibodies. We found that SUMO1 and SUMO2/3 localize partially with the synaptic markers PSD95 and synaptophysin, confirming previously published evidence indicating the presence of SUMO proteins at the synapse. Moreover, we determined that Ubc9 partially colocalizes with PSD95 and to a less extent with synaptophysin, alone or together with SUMO1 and SUMO2/3.

## Materials and Methods

### Primary Cultures

Hippocampal neurons were prepared from two-day-old CD1 mice as described (Restelli et al., 2010). Briefly, dissected hippocampi were incubated in 5.8 mM MgCl_2_, 0.5 mM CaCl_2_, 3.2 mM HEPES, 0.2 mM NaOH (pH 7.4, 292 mOsm) containing 20 U/mL papain (Sigma) at 37°C for 30 min. Trypsin inhibitor (Sigma) was added and the tissues were incubated for 45 min at room temperature. Next, the tissues were mechanically dissociated by passing through P1000 pipette filtered tip. Cells were plated at 75–100,000 cells/well on poly-L-lysine-coated (25 μg/mL) plates and maintained in Neurobasal Basal Medium (Gibco) supplemented with B27 (Gibco), penicillin/streptomycin and glutamine 2 mM. To reduce the number of non-neuronal cells, cytosine arabinoside (araC, final concentration 10 μM, Sigma) was added to the medium 4 days after plating. MitoTracker^TM^ Deep Red FM (Thermo Fisher Scientific, #M22426) was given to primary neurons for 20 min before PFA fixation at a final concentration of 50 nM.

### Animals

Procedures involving animals were conducted in conformity with the institutional guidelines at the Istituto di Ricerche Farmacologiche Mario Negri IRCCS, in compliance with national (D.lgs 26/2014; Authorization n. 19/2008-A issued March 6, 2008 by Ministry of Health) and international laws and policies (EEC Council Directive 2010/63/UE; the NIH Guide for the Care and Use of Laboratory Animals, 2011 edition). They were reviewed and approved by the Mario Negri Institute Animal Care and Use Committee, which includes *ad hoc* members for ethical issues, and by the Italian Ministry of Health (Decreto no. 420/2017-PR). Animal facilities meet international standards and are regularly checked by a certified veterinarian who is responsible for health monitoring, animal welfare supervision, experimental protocols, and review of procedures.

### SUMO Antibody Production

Custom SUMO 1 and SUMO2 antibody were made as previously described (Matsuzaki et al., 2015). Briefly, a peptide corresponding to SUMO1 N-terminal residues C-terminus residues 73–97 (IADNHTPKELGMEEEDVIEVYQEQT) and a peptide corresponding to SUMO2 N-terminal residues 3–24 (EEKPKEGVKTENDHINLKVAGQ) were chemically synthesized and used for polyclonal antibody generation in rabbits. The antigen used to raise the SUMO2 polyclonal antibody differs from SUMO3 by only 2 amino acids and likely recognizes SUMO3 as well.

### Immunofluorescence Experiments

Primary neurons were cultured in Ibidi μ-Slide 8 Well plates and immunolabeled at 12–18 days *in vitro* (DIV). Immunofluorescence experiments were performed adapting the protocol described in Daniel et al. (2017). Briefly, primary neurons were fixed in 4% paraformaldehyde for 15 min, then permeabilized in PBS with 0.2% Triton X-100 for 1 min. For the detection of extranuclear SUMO proteins, two protocols have been described. One uses digitonin as the permeabilizing detergent and the second one Triton X-100. While the latter is often used in immunofluorescence procedures, digitonin is mainly used to selectively permeabilize the plasma membrane and not the nuclear one. The rationale behind the use of digitonin is that since SUMO proteins are mainly nuclear, avoiding the permeabilization of the nuclear membrane allows for enhanced detection of SUMO proteins outside the nuclei (Girach et al., 2013; Jaafari et al., 2013; Craig et al., 2015; Daniel et al., 2017). We, however, opted to use Triton X-100 since we were able to detect both nuclear and extranuclear signal for SUMO2/3 with our instrument. Neurons were blocked in 1% BSA in PBS for 1 h and incubated for 2 h with primary antibodies at room temperature in 1% BSA in PBS with 0.2% Triton X-100. The primary antibodies used were custom anti-SUMO2/3 and anti-SUMO1 (Matsuzaki et al., 2015); NeuN and Map2 (2.5 μg/mL Merck Millipore); SUMO2/3 (4 μg/mL Abcam, #196278. Immunogen: recombinant full length protein corresponding to Human Sumo 2 amino acids 2-95), SUMO2/3 (4 μg/mL Cell Signaling, #18H8. Immunogen: synthetic peptide from the amino terminus of human SUMO2/3 with epitope centered at aminoacid 7), PSD95 (4 μg/mL NeuroMab clone K28/43), synaptophysin (4 μg/mL Sigma, #S5768), Ubc9 (2 μg/mL Abcam, ab21193), Ubc9 (4 μg/mL Abcam, ab33044). Secondary antibodies were added for 1 h (Thermo Fisher Scientific DyLight^TM^ Fluor secondary antibodies). Nuclear staining was obtained with Hoechst 33342 (Thermo Fisher Scientific) at the concentration of 1 μg/mL. Cells were next mounted using ProLong^TM^ Diamond Antifade Mountant (Thermo Fisher Scientific).

### SUMO2/3 Antibody Neutralization

SUMO2/3 custom antibody was neutralized by incubating it for 1 h at room temperature in 1% BSA in PBS with five times excess of recombinant human SUMO2 full-length protein (UL-752, Boston Biochem). We next performed the staining protocol described above using the blocked antibody.

### Confocal and Structured Illumination Microscopy (SIM)

Microscopy techniques were adapted from protocols previously described (Violatto et al., 2019). Briefly, samples were acquired using Nikon A1 Confocal and Nikon N-SIM microscopes. Confocal images were collected using 40× objectives with a stack thickness of 0.48 μm. For N-SIM super resolution acquisitions, a CFI SR HP Apochromat TIRF 100XC oil objective was used and images were acquired in 3D-SIM mode with a stack thickness of 0.12 μm. Images were processed with Fiji (ImageJ) software.

### Statistical Analysis

To assess overlapping fluorescence signal, colocalization analysis were carried out using JACoP plug-in (ImageJ). 40 SIM images for each condition, at a resolution of 100 nm, were analyzed. The images were taken from four independent experiments. Pearson’s correlation coefficient was used to describe the overall overlap of signals and Mander’s M1 and M2 were chosen as parameters to describe reciprocal colocalization between SUMO2/3, SUMO1, and Ubc9 and the synaptic markers synaptophysin or PSD95. For Mander’s analysis manual threshold was set to avoid background contribution. Graphs were obtained with GraphPad Prism 7.

## Results

### SUMO2/3 Is Found Predominantly in the Nuclei of Cultured Hippocampal Neurons

To determine the localization of SUMO2/3 in cultured hippocampal primary neurons we used three-color confocal microscopy. We imaged endogenous SUMO2/3 using a custom-made antibody against the protein (Matsuzaki et al., 2015) along with two neuronal markers, NeuN and Map2. Similarly to what has been found in other types of cells (Gareau and Lima, 2010), SUMO2/3 localizes mainly in the nucleus (Supplementary Figure S1A). A caveat of our study is the possible off-target binding of our custom-made antibody that can significantly affect the interpretation of the results. To control for this, a solution is the use of primary neurons obtained from knock-out mice as a negative control, as done by Daniel et al. (2017) for SUMO1. Unfortunately, SUMO2 knock-out mice are embryonic lethal (Wang et al., 2014) and cannot therefore be used for this analysis. To address this, we used two additional commercial SUMO2/3 antibodies raised against different epitopes of the protein (Supplementary Figures S1B, S5A). During the analysis however, we also observed a weak extranuclear signal in neurons. This was seen with all three antibodies (Supplementary Figures S1, S4A).

### SUMO2/3 Partially Colocalizes With the Presynaptic Marker Synaptophysin

**FIGURE 1:**
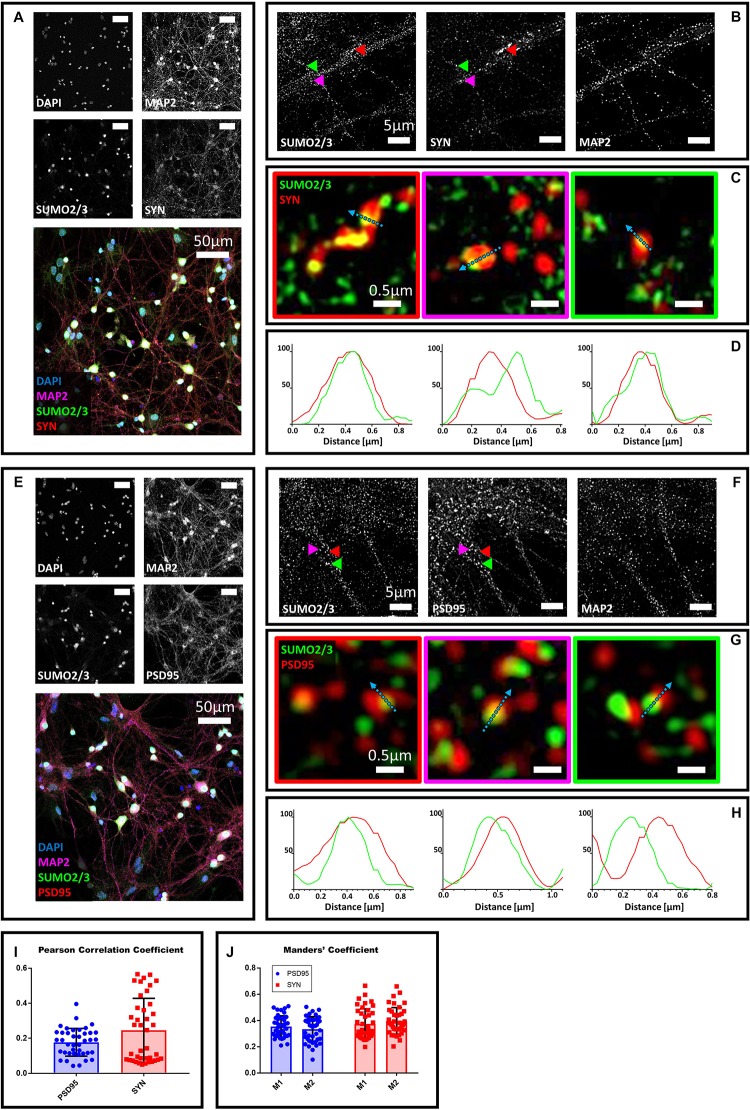
Confocal microscopy and SIM analyses of DIV18 primary hippocampal neurons to determine the localization of SUMO2/3, synaptophysin and PSD95. **(A)** Confocal microscopy of primary neurons. Cells were immunostained for SUMO2/3 using our custom antibody (green), synaptophysin (red) and Map2 (magenta). DAPI was used to stain the nuclei. Scale bar, 50 μm. Images were obtained using a 40× objective and displayed as *z* projection. **(B)** SIM analysis using a 100× objective. Colored arrowheads indicate the position of the inset shown in panel **(C)**. **(C)** The Merge images represent single stack of SUMO2/3 (green) and synaptophysin (red). Scale bar, 0.5 μm. **(D)** Intensity profile normalized for each channel to 100 (arbitrary unit) using the same color code of SIM merge images and representing the values indicated by the cyan arrow. **(E)** Confocal microscopy of primary neurons. Cells were immunostained for SUMO2/3 (custom antibody, green), PSD95 (red) and Map2 (magenta). DAPI was used to stain the cell nuclei. Scale bar, 50 μm. Images were obtained using a 40× objective and displayed as *z* projection. **(F)** SIM analysis using a 100× objective with colored arrows that indicate the position of the inset shown in panel **(G)**. **(G)** Merge channel represent single stack image of SUMO2/3 (green) and PSD95 (red). Scale bar, 0.5 μm. **(H)** Intensity profile normalized for each channel to 100 (arbitrary unit) using the same color code of SIM merge images and representing the values indicated by the cyan arrow. **(I)** Pearson Correlation Coefficient between SUMO2/3 (custom antibody) and PSD95 (blue) and synaptophysin (SYN) (red). **(J)** SUMO2/3 fraction that colocalizes with PSD95 or synaptophysin (SYN) (M1) and PSD95 or synaptophysin fraction that colocalizes with SUMO2/3 (M2). Date are the mean ± SD of 40 fields from four independent experiments.

To determine whether the extranuclear SUMO2/3 may localize at synapses, we co-stained primary hippocampal neurons with SUMO2/3 and the presynaptic marker synaptophysin, a conserved vesicle membrane protein (Wiedenmann and Franke, 1985). We first visualized large portions of the field to assess neuronal morphology using confocal microscopy and a 40× objective (Figure 1A). Next, we used structure illumination microscopy (SIM) (Gustafsson, 2000). This super resolution microscopy technique improves the resolution of conventional microscopes from 250 nm to about 100 nm, making it better suited to study protein localization in the narrow areas of synapses, which are between 0.03 and 0.15 μm in size (Papa et al., 1995; Schikorski and Stevens, 1997; Huang et al., 2009; Igarashi et al., 2018). We found that SUMO2/3 partially colocalizes with synaptophysin (Figures 1A–D), thus suggesting that SUMO2/3 may be present in presynaptic terminals. We used Mander’s coefficients to assess colocalization between SUMO2/3 and synaptophysin, allowing independent measures of the fraction of SUMO2/3 that overlaps with synaptophysin signal (M1), and the fraction of synaptophysin that overlaps with SUMO2/3 signal (M2) (Dunn et al., 2011). We obtained values above 0 and below 0.5, indicating a low-to-medium colocalization rate. We also used Pearson correlation coefficient to assess the overall colocalization. Pearson’s correlation coefficients were below 0.5, indicating low to medium colocalization (Figures 1I,J). As a control, we neutralized the SUMO2/3 custom antibody with recombinant human SUMO2/3 protein. The neutralized antibody did not detect any signal colocalizing with synaptophysin (Supplementary Figure S2). We confirmed the results with two additional commercially available antibodies (Supplementary Figures S3A–D, S4A–D).

### SUMO2/3 Partially Colocalizes With the Postsynaptic Marker PSD95

To determine whether SUMO2/3 was also present at the postsynaptic compartment, we co-stained neurons with the postsynaptic marker PSD95 (Hunt et al., 1996). We found that SUMO2/3 partially colocalizes with PSD95, suggesting that SUMO2/3 may be present at postsynaptic sites (Figures 1E–H). We confirmed these results by neutralizing the SUMO2/3 custom antibody (Supplementary Figure S2) and by using two additional commercially available SUMO2/3 antibodies (Supplementary Figures S3E–H, 4E–H). We quantified the colocalization of SUMO2/3 with PSD95 using Mander’s coefficients and Pearson correlation coefficient (Figures 1I,J). Values were indicative of a low-to-medium colocalization (Table 1).

**TABLE 1:** Summary of Mander’s coefficient values and Pearson’s correlation coefficients.

	**UBC9+SYN**	**UBC9+PSD95**	**SUMO2/3+SYN**	**SUMO2/3+SD95**	**SUMO1+SYN**	**SUMO1+SD95**
Pearson’s	0.0953 ± 0.03301	0.4047 ± 0.2027	0.2456 ± 0.1827	0.1775 ± 0.07899	0.07788 ± 0.04083	0.1487 ± 0.0.703
M1	0.277 ± 0.066	0.590 ± 0.119	0.373 ± 0.116	0.355 ± 0.081	0.245 ± 0.065	0.307 ± 0.098
M2	0.183 ± 0.061	0.387 ± 0.151	0.396 ± 0.102	0.344 ± 0.95	0.329 ± 0.072	0.306 ± 0.124

A possible caveat of this analysis is the confounding presence of mitochondria in dendrites. Synapses are enriched in mitochondria and SUMOylation helps to regulate mitochondrial function. To determine whether the presence of SUMO2/3 at the synapse correlates with mitochondria, we used a mitochondria dye, MitoTracker, to co-stain mitochondria with SUMO2/3 and PSD95 or synaptophysin. We found that SUMO2/3 partially colocalizes with PSD95 and synaptophysin with or without mitochondria (Supplementary Figure S5). Thus SUMO2/3 can be at pre- and postsynaptic markers positive loci independently of mitochondria.

### Ubc9 Partially Colocalizes With Synaptophysin and PSD95 Alone or Together With SUMO1 and SUMO2/3

**FIGURE 2:**
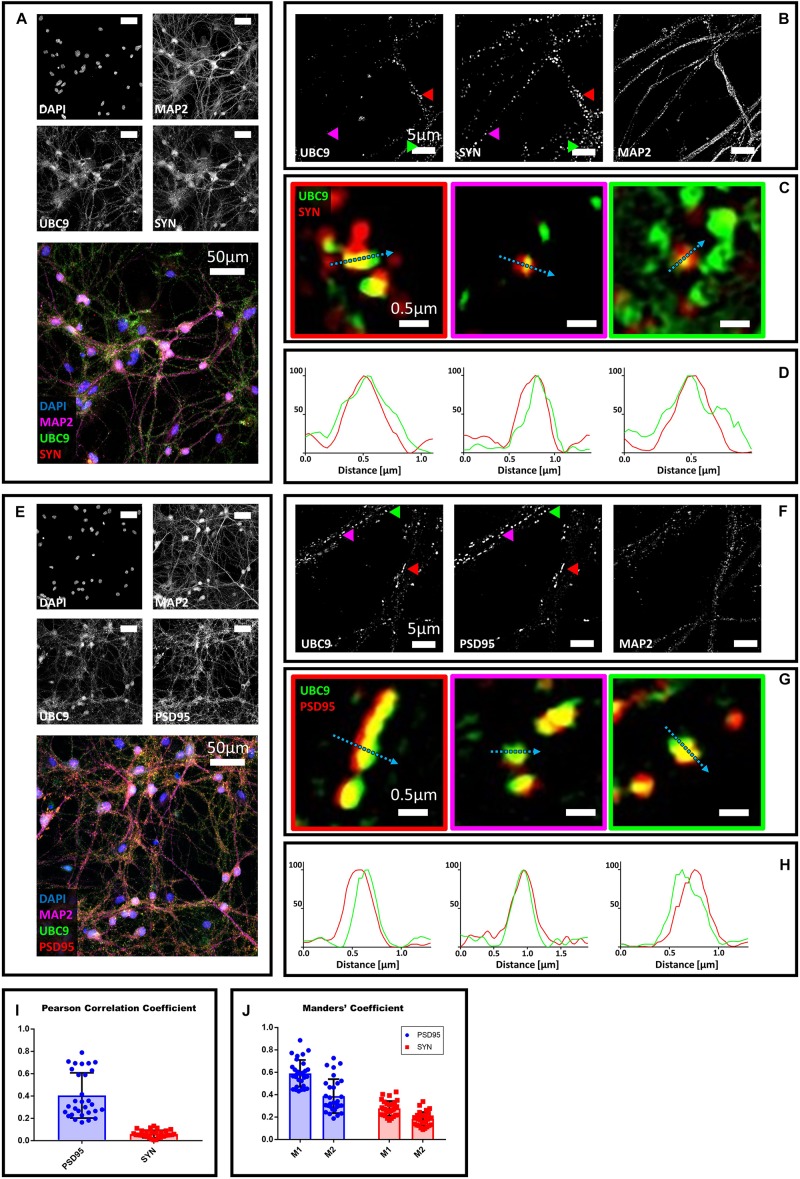
Confocal microscopy and SIM analyses of DIV18 primary hippocampal neurons to determine the localization of Ubc9, synaptophysin and PSD95. **(A)** Confocal microscopy of primary neurons. Cells were immunostained for Ubc9 (Abcam #ab33044, green), synaptophysin (red) and Map2 (magenta). DAPI was used to stain the nuclei. Scale bar, 50 μm. Images were obtained using a 40× objective and displayed as *z* projection. **(B)** Colored arrowheads indicate the position of the inset shown in panel **(C)**. **(C)** Merge images represent single stack of Ubc9 (green) and synaptophysin (red). Scale bar, 0.5 μm. **(D)** Intensity profile normalized for each channel to 100 (arbitrary unit) using the same color code of SIM merge images and representing the values indicated by the cyan arrow. **(E)** Confocal microscopy of primary neurons. Cells were immunostained for Ubc9 (custom antibody, green), PSD95 (red) and Map2 (magenta). DAPI was used to stain the cell nuclei. Scale bar, 50 μm. Images were obtained using a 40× objective and displayed as *z* projection. **(F)** SIM microscopy, colored arrows indicate the position of the inset shown in panel **(G)**. **(G)** Merge channel represent single stack image of Ubc9 (green) and PSD95 (red). Scale bar, 0.5 μm. **(H)** Intensity profile normalized for each channel to 100 (arbitrary unit) using the same color code of SIM merge images and representing the values indicated by the cyan arrow. **(I)** Pearson Correlation Coefficient between Ubc9 (#ab33044) and PSD95 (blue) and synaptophysin (SYN) (red). **(J)** Ubc9 fraction that colocalizes with PSD95 or synaptophysin (SYN) (M1) and PSD95 or synaptophysin fraction that colocalizes with Ubc9 (M2). Date are the mean ± SD of 40 fields from four independent experiments.

**FIGURE 3:**
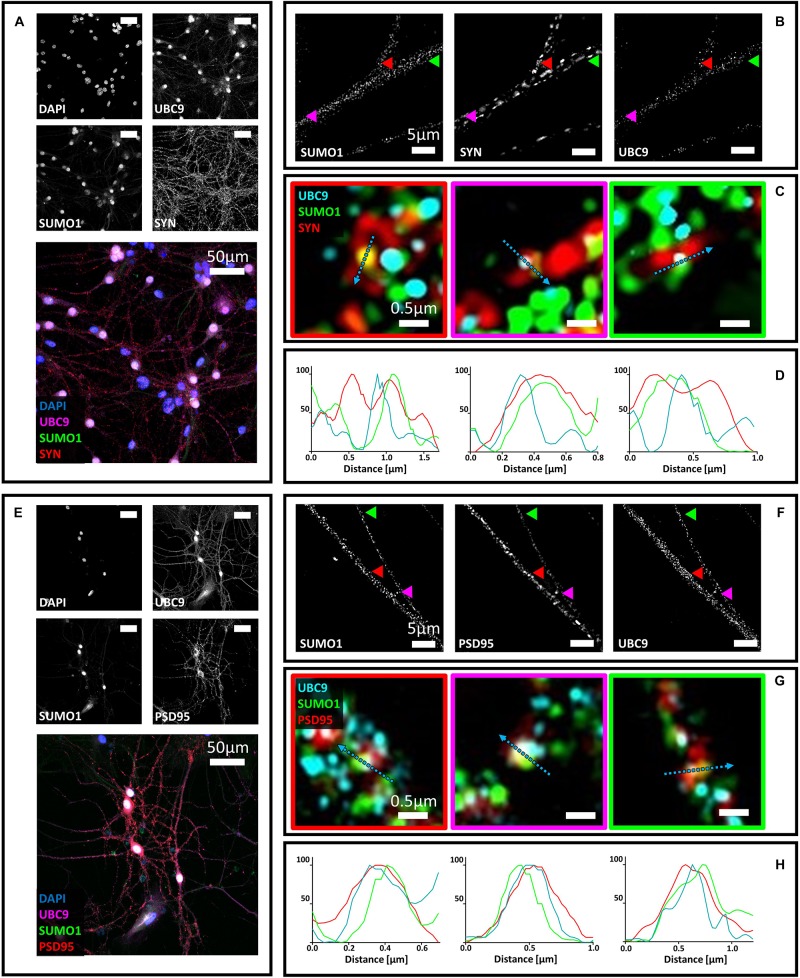
Confocal microscopy and SIM analyses of primary hippocampal neurons to determine the distribution of Ubc9, SUMO1, SUMO2/3, synaptophysin, and PSD95. **(A)** Confocal microscopy of primary neurons. Cells were immunostained for SUMO1 (custom antibody, green), synaptophysin (red) and Ubc9 (Abcam #ab21193, magenta). DAPI was used to stain the nuclei. Scale bar, 50 μm. Images were obtained using a 40× objective and displayed as *z* projection. **(B)** SIM microscopy. Colored arrowheads indicate the position of the inset shown in panel **(C)**. **(C)** Merge images represent single stack of SUMO1 (green), synaptophysin (red) and Ubc9 (cyan). Scale bar, 0.5 μm. **(D)** Intensity profile normalized for each channel to 100 (arbitrary unit) using the same color code of SIM merge images and representing the values indicated by the cyan arrow. **(E)** Confocal microscopy of primary neurons. Cells were immunostained for SUMO1 (custom antibody, green), PSD95 (red), and Ubc9 (Abcam #ab21193, magenta). DAPI was used to stain the cell nuclei. Scale bar, 50 μm. Images were obtained using a 40× objective and displayed as *z* projection. **(F)** SIM analysis using a 100× objective with colored arrows that indicate the position of the inset shown in panel **(G)**. **(G)** Merge images represent single stack of SUMO1 (green), PSD95 (red) and Ubc9 (cyan). Scale bar, 0.5 μm. **(H)** Intensity profile normalized for each channel to 100 (arbitrary unit) using the same color code of SIM merge images and representing the values indicated by the cyan arrow.

**FIGURE 4:**
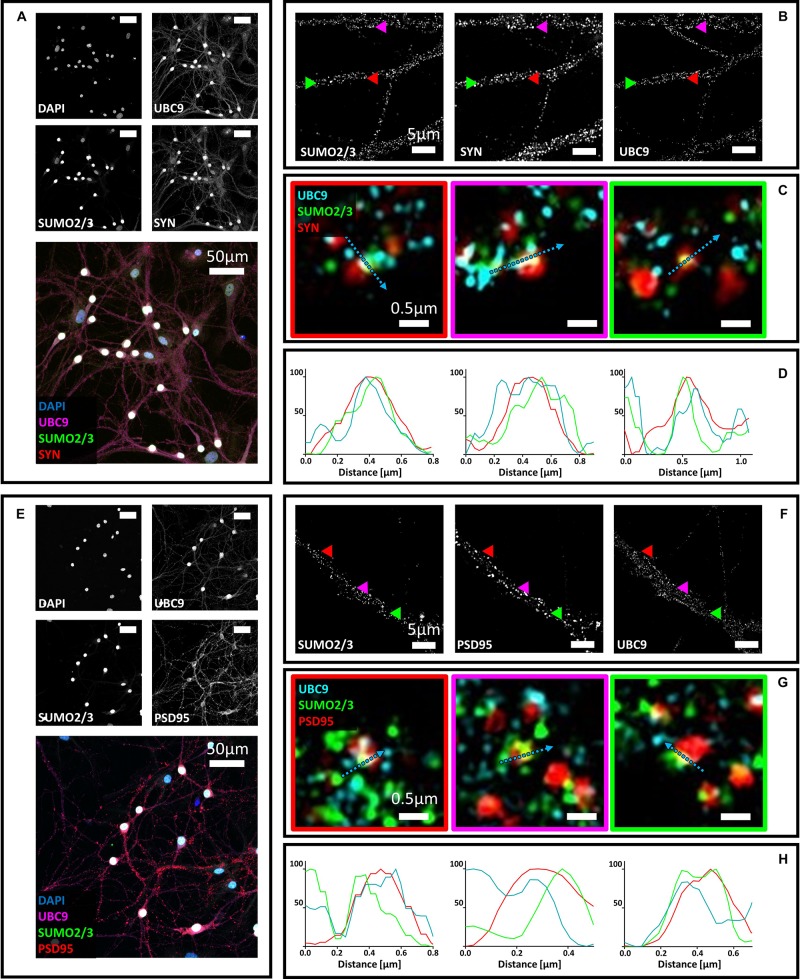
**(A)** Confocal microscopy of primary neurons. Cells were immunostained for SUMO2/3 (custom antibody, green), synaptophysin (red) and Ubc9 (Abcam #ab21193, magenta). DAPI was used to stain the nuclei. Scale bar, 50 μm. Images were obtained using a 40× objective and displayed as *z* projection. **(B)** SIM microscopy. Colored arrowheads indicate the position of the inset shown in panel **(C)**. **(C)** Merge images represent single stack of SUMO2/3 (green), synaptophysin (red) and Ubc9 (cyan). Scale bar, 0.5 μm. **(D)** Intensity profile normalized for each channel to 100 (arbitrary unit) using the same color code of SIM merge images and representing the values indicated by the cyan arrow. **(E)** Confocal microscopy of primary neurons. Cells were immunostained for SUMO2/3 (custom antibody, green), PSD95 (red) and Ubc9 (Abcam #ab21193, magenta). DAPI was used to stain the cell nuclei. Scale bar, 50 μm. Images were obtained using a 40× objective and displayed as *z* projection. **(F)** SIM microscopy, colored arrows indicate the position of the inset shown in panel **(G)**. **(G)** Merge images represent single stack of SUMO2/3 (green), PSD95 (red), and Ubc9 (cyan). Scale bar, 0.5 μm. **(H)** Intensity profile normalized for each channel to 100 (arbitrary unit) using the same color code of SIM merge images and representing the values indicated by the cyan arrow.

Next, we studied the localization of Ubc9 in primary neurons with synaptic markers. We co-stained hippocampal neurons with Ubc9 and PSD95 or synaptophysin. We found that Ubc9 partially colocalizes with both pre- and postsynaptic markers, indicating that not only SUMO2/3 but also Ubc9 may be present at the synapse (Figure 2). Quantitative analysis indicated a medium-high colocalization rate for Ubc9 and PSD95 and a low colocalization rate for Ubc9 and synaptophysin (Figures 2I,J). To determine whether Ubc9 co-stains with pre- and postsynaptic markers together with SUMO proteins, we first studied, with two different antibodies, whether SUMO1 could also partially localize with PSD95 and synaptophysin. Similarly to SUMO2/3, we found that SUMO1 is predominately a nuclear protein that partially localizes with the two synaptic markers (Supplementary Figures S6–S8). Quantitative analysis of the colocalization of SUMO1 with PSD95 and synaptophysin indicated a lower colocalization rate compared to SUMO2/3 (Supplementary Figures S7I,J and Table 1). Like SUMO2/3, also SUMO1 partially colocalized with PSD95 and synaptophysin independently of mitochondria (Supplementary Figure S9). We next used another antibody against Ubc9, raised in goat, to determine whether SUMO proteins localized with Ubc9 at PSD95 and synaptophysin positive loci. We confirmed that Ubc9 localizes with both PSD95 and synaptophysin, and it partially does so with SUMO2/3 and SUMO1 (Figures 3, 4).

## Discussion

Since the first reports of the presence of SUMO proteins at the synapse (Martin et al., 2007), most studies focused on SUMO1 (Henley et al., 2018). This variant is considered to be the prototypical SUMO protein and it is the most studied. However, there are significant functional differences between the different SUMO variants, as highlighted by the partial overlap of target proteins (Saitoh and Hinchey, 2000; Vertegaal et al., 2006). While in recent years the extranuclear function of SUMO1 in neurons has been placed under scrutiny, less attention has been paid to the presence of SUMO2/3 or SUMOylation enzymes at the synapse. Due to the prominent role attributed to SUMOylation in neurons, in both physiological and pathological conditions (Lee et al., 2013, 2014; Henley et al., 2018), understanding whether SUMO proteins, and SUMOylation enzymes are present at the synapse is of crucial interest (Wilkinson et al., 2017; Daniel et al., 2018). In order to confirm whether SUMO2/3 and the E2 SUMOylation enzyme Ubc9 are present at synaptic sites (Li et al., 2004), we first sought to determine their distribution within neuronal cells. In the present study, we found that three antibodies raised against different parts of the SUMO2/3 protein show extranuclear staining that partially colocalizes with synaptic markers in primary hippocampal neurons. We also determined that the E2 SUMO ligase Ubc9 partially colocalizes with PSD95 and synaptophysin, with or without SUMO proteins.

### SUMO Proteins and Enzymes at the Synapse

We detected SUMO1, SUMO2/3, and Ubc9 in both pre- and post-synaptic compartments, but the colocalization with the specific markers we used is only partial, as shown by Pearson’s and Mander’s values. The majority of PSD95 and synaptophysin positive structures are not positive for SUMO proteins, suggesting that only a portion of pre- and post-synaptic sites contain SUMO1, SUMO2/3 or Ubc9. The presence of SUMO1 and SUMO2/3 signal at the synapse may represent both free SUMO available for conjugation and/or SUMO-conjugated synaptic proteins. In 2015, we showed that the protein CPEB3 is SUMOylated by SUMO2 in synaptosomal preparations and that SUMO2 conjugation is crucial for the function of this translational regulator implicated in long-term memory storage (Drisaldi et al., 2015; Fioriti et al., 2015). The following year, Ghosh et al. (2016) showed that SUMOylation by SUMO2 is among the several post-translational modifications that regulate GABAergic transmission. Moreover, the SUMOylation enzymes SAE1 and Ubc9 are enriched in dendritic spines, suggesting the presence of the SUMOylation machinery and active SUMOylation at the synapse (Loriol et al., 2012, 2014; Schorova and Martin, 2016). However, it is also possible that SUMOylation of target proteins occurs outside the synapse and SUMOylation by SUMO1 or SUMO2/3 may function as a translocation signal, similarly to SUMOylation by SUMO1 of the RNA-binding protein La (van Niekerk et al., 2007).

### Mitochondria, SUMO Proteins, and Synapses

Since SUMO proteins regulate mitochondria functions, we investigated whether SUMO2/3 and SUMO1 colocalized with mitochondria at pre- and postsynaptic sites. We found that SUMO proteins localize at the synapse independently of mitochondria, suggesting that they may regulate synaptic activity and not only synaptic mitochondria function.

### Future Directions

There are a number of questions regarding the role of SUMO isoforms and SUMOylatino enzymes at the synapse that need to be addressed. We still do not know whether the presence of SUMO variants at the synapse is dynamic and may change with synaptic activity, similarly to what has been described for Ubc9 (Loriol et al., 2014). It is also unclear what is the role of SUMOylation during pathologies that affect the synapses, although recent work suggests a possible involvement in Alzheimer’s disease (Lee et al., 2013, 2014; Maruyama et al., 2018). More functional studies are therefore required to fully uncover the role of SUMO at the synapse.

## Data Availability Statement

The datasets generated for this study are available on request to the corresponding author.

## Ethics Statement

The animal study was reviewed and approved by the Mario Negri Institute Animal Care and Use Committee.

## Author Contributions

LC and LF designed the work and drafted the manuscript. LC, LR, CN, ER, and AC performed the experiments and analyzed the data. All authors reviewed and edited the manuscript, and approved the final version of the manuscript and agreed to be accountable for all aspects of the work regarding questions related to the accuracy or integrity of any part of the work.

## Conflict of Interest

The authors declare that the research was conducted in the absence of any commercial or financial relationships that could be construed as a potential conflict of interest.

## Supplementary Material

FIGURE S1 Confocal microscopy of DIV18 primary hippocampal neurons. **(A)** Neurons were immunostained by SUMO2/3 (custom antibody; green), NeuN (red), and Map2 (magenta). DAPI (blue) was used to stain the nuclei. Scale bar, 50 μm. Images were obtained using a 40× objective and displayed as *z* projection. **(B)** Cells were stained with a commercial anti-SUMO2/3 antibody (Abcam #193267; green), NeuN (red), and Map2 (magenta). DAPI was used to stain the nuclei. Scale bar, 50 μm. Images were obtained using a 40× objective and displayed as *z* projection.

FIGURE S2 SIM analysis of primary hippocampal neurons stained with neutralized SUMO2/3 antibody. Cells were immunostained by neutralized SUMO2/3 (custom antibody), synaptophysin, PSD95 and Map2.

FIGURE S3 Confocal microscopy and SIM analyses of DIV18 primary hippocampal neurons to determine the localization of SUMO2/3, synaptophysin and PSD95. **(A)** Confocal microscopy of primary neurons. Cells were immunostained for SUMO2/3 (Abcam #193267, green), synaptophysin (red) and Map2 (magenta). DAPI was used to stain the nuclei. Scale bar, 50 μm. Images were obtained using a 40× objective and displayed as *z* projection. **(B)** SIM analysis using a 100× objective. Colored arrowheads indicate the position of the inset shown in panel **(C)**. **(C)** The Merge images represent single stack of SUMO2/3 (green) and synaptophysin (red). Scale bar, 0.5 μm. **(D)** Intensity profile normalized for each channel to 100 (arbitrary unit) using the same color code of SIM merge images and representing the values indicated by the cyan arrow. **(E)** Confocal microscopy of primary neurons. Cells were immunostained for SUMO2/3 (Abcam #193267, green), PSD95 (red), and Map2 (magenta). DAPI was used to stain the cell nuclei. Scale bar, 50 μm. Images were obtained using a 40× objective and displayed as *z* projection. **(F)** SIM analysis using a 100× objective with colored arrows that indicate the position of the inset shown in panel **(G)**. **(G)** Merge channel represent single stack image of (Abcam #193267, green) and PSD95 (red). Scale bar, 0.5 μm. **(H)** Intensity profile normalized for each channel to 100 (arbitrary unit) using the same color code of SIM merge images and representing the values indicated by the cyan arrow.

FIGURE S4 Confocal microscopy and SIM analyses of DIV18 primary hippocampal neurons to determine the localization of SUMO2/3, PSD95 and synaptophysin. **(A)** Confocal microscopy of primary neurons. Cells were immunostained by SUMO2/3 (cell signaling #18H8, green), synaptophysin (red) and Map2 (magenta). DAPI was used to stain the nuclei. Scale bar, 50 μm. Images were obtained using a 40× objective and displayed as *z* projection. **(B)** SIM analysis using a 100× objective with colored arrows that indicate the position of the inset shown in panel **(C)**. **(C)** Merge channel represent single stack image of SUMO2/3 (green) and synaptophysin (red). Scale bar, 0.5 μm. **(D)** Intensity profile normalized for each channel to 100 (arbitrary unit) using the same color code of SIM merge images and representing the values indicated by the cyan arrow. **(E)** Confocal microscopy of primary neurons. Cells were immunostained by SUMO2/3 (cell signaling #18H8, green), PSD95 (red) and Map2 (magenta). DAPI was used to stain the nuclei. Scale bar, 50 μm. Images were obtained using a 40× objective and displayed as *z* projection. **(F)** SIM analysis using a 100× objective with colored arrows that indicate the position of the inset shown in panel **(G)**. **(G)** Merge channel represent single stack image of SUMO2/3 (green) and PSD95 (red). Scale bar, 0.5 μm. **(H)** Intensity profile normalized for each channel to 100 (arbitrary unit) using the same color code of SIM merge images and representing the values indicated by the cyan arrow.

FIGURE S5 Confocal microscopy and SIM analyses of primary hippocampal neurons to determine the localization of SUMO2/3, synaptic markers and mitochondria. **(A,B)** Confocal microscopy of primary neurons. Cells were immunostained for SUMO2/3 using our custom antibody (green), mitochondria (MITO) (red) and synaptophysin (SYN) or PSD95 (magenta). DAPI was used to stain the nuclei. Scale bar, 50 μm. Images were obtained using a 40× objective and displayed as *z* projection. **(C,D)** SIM analysisusing a 100× objective. Colored arrowheads indicate the position of the inset. Merge images represent single stack of SUMO2/3 (green), synaptophysin (SYN) or PSD95 (cyan) and mitochondria (MITO) (red). Scale bar, 0.5 μm. Intensity profile normalized for each channel to 100 (arbitrary unit) using the same color code of SIM merge images and representing the values indicated by the cyan arrow. **(E)** SIM analysis using a 100× objective. White square indicates the inset. Merge image of soma represents single stack of SUMO2/3 (green) and mitochondria (MITO) (red). Scale bars, 5 and 0.5 μm, respectively. Intensity profile normalized for each channel to 100 (arbitrary unit) using the same color code of SIM merge images and representing the values indicated by the cyan arrow.

FIGURE S6 Confocal microscopy of primary hippocampal neurons. Neurons were immunostained by SUMO1 (custom antibody; green), NeuN (red) and Map2 (magenta). DAPI (blue) was used to stain the nuclei. Scale bar, 50 μm. Images were obtained using a 40× objective and displayed as *z* projection.

FIGURE S7 Confocal microscopy and SIM analyses of DIV18 primary hippocampal neurons to determine the localization of SUMO1 and synaptophysin. **(A)** Confocal microscopy of primary neurons. Cells were immunostained for SUMO1 using our custom antibody (green), synaptophysin (red) and Map2 (magenta). DAPI was used to stain the nuclei. Scale bar, 50 μm. Images were obtained using a 40× objective and displayed as *z* projection. **(B)** SIM analysis using a 100× objective. Colored arrowheads indicate the position of the inset shown in panel **(C)**. **(C)** The Merge images represent single stack of SUMO1 (green) and synaptophysin (red). Scale bar, 0.5 μm. **(D)** Intensity profile normalized for each channel to 100 (arbitrary unit) using the same color code of SIM merge images and representing the values indicated by the cyan arrow. **(E)** Confocal microscopy of primary neurons. Cells were immunostained for SUMO1 (Abcam, green), synaptophysin (red) and Map2 (magenta). DAPI was used to stain the nuclei. Scale bar, 50 μm. Images were obtained using a 40× objective and displayed as *z* projection. **(F)** SIM analysis using a 100× objective. Colored arrowheads indicate the position of the inset shown in panel **(G)**. **(G)** The Merge images represent single stack of SUMO1 (green) and synaptophysin (red). Scale bar, 0.5 μm. **(H)** Intensity profile normalized for each channel to 100 (arbitrary unit) using the same color code of SIM merge images and representing the values indicated by the cyan arrow. **(I)** Pearson Correlation Coefficient between SUMO1 (custom antibody) and PSD95 (blue) and synaptophysin (SYN) (red). **(J)** SUMO1 fraction that colocalizes with PSD95 or synaptophysin (SYN) (M1) and PSD95 or synaptophysin fraction that colocalizes with SUMO1 (M2). Date are the mean ± SD of 40 fields from four independent experiments.

FIGURE S8 Confocal microscopy and SIM analyses of primary hippocampal neurons to determine the localization of SUMO1 and PSD95. **(A)** Confocal microscopy of primary neurons. Cells were immunostained for SUMO1 using our custom antibody (green), PSD95 (red) and Map2 (magenta). DAPI was used to stain the nuclei. Scale bar, 50 μm. Images were obtained using a 40× objective and displayed as *z* projection. **(B)** SIM analysis using a 100× objective. Colored arrowheads indicate the position of the inset shown in panel **(C)**. **(C)** The Merge images represent single stack of SUMO1 (green) and PSD95 (red). Scale bar, 0.5 μm. **(D)** Intensity profile normalized for each channel to 100 (arbitrary unit) using the same color code of SIM merge images and representing the values indicated by the cyan arrow. **(E)** Confocal microscopy of primary neurons. Cells were immunostained for SUMO1 (Abcam, green), PSD95 (red) and Map2 (magenta). DAPI was used to stain the nuclei. Scale bar, 50 μm. Images were obtained using a 40× objective and displayed as *z* projection. **(F)** SIM analysis using a 100× objective. Colored arrowheads indicate the position of the inset shown in panel **(G)**. **(G)** The Merge images represent single stack of SUMO1 (green) and PSD95 (red). Scale bar, 0.5 μm. **(H)** Intensity profile normalized for each channel to 100 (arbitrary unit) using the same color code of SIM merge images and representing the values indicated by the cyan arrow.

FIGURE S9 Confocal microscopy and SIM analyses of primary hippocampal neurons to determine the localization of SUMO1, synaptic markers and mitochondria. **(A,B)** Confocal microscopy of primary neurons. Cells were immunostained for SUMO1 using our custom antibody (green), mitochondria (MITO) (red) and synaptophysin (SYN) or PSD95 (magenta). DAPI was used to stain the nuclei. Scale bar, 50 μm. Images were obtained using a 40× objective and displayed as *z* projection. **(C,D)** SIM analysis using a 100× objective. Colored arrowheads indicate the position of the inset. Merge images represent single stack of SUMO1 (green), synaptophysin (SYN) or PSD95 (cyan) and mitochondria (MITO) (red). Scale bar, 0.5 μm. Intensity profile normalized for each channel to 100 (arbitrary unit) using the same color code of SIM merge images and representing the values indicated by the cyan arrow. **(E)** SIM analysis using a 100× objective. White square indicates the inset. Merge image of soma represents single stack of SUMO1 (green) and mitochondria (MITO) (red). Scale bars, 5 and 0.5 μm, respectively. Intensity profile normalized for each channel to 100 (arbitrary unit) using the same color code of SIM merge images and representing the values indicated by the cyan arrow.
